# Recipient HLA-G +3142 CC Genotype and Concentrations of Soluble HLA-G Impact on Occurrence of CMV Infection after Living-Donor Kidney Transplantation

**DOI:** 10.3390/ijms18112338

**Published:** 2017-11-05

**Authors:** Hana Guberina, Rafael Tomoya Michita, Sebastian Dolff, Anja Bienholz, Mirko Trilling, Falko M. Heinemann, Peter A. Horn, Andreas Kribben, Oliver Witzke, Vera Rebmann

**Affiliations:** 1 Department of Infectious Diseases, University Hospital Essen, University Duisburg-Essen, 45147 Essen, Germany; Sebastian.Dolff@uk-essen.de (S.D.); Oliver.Witzke@uk-essen.de (O.W.); 2 Institute for Transfusion Medicine, University Hospital Essen, University Duisburg-Essen, 45147 Essen, Germany; Rafael.Tomoya-Michita@uk-essen.de (R.T.M.); Falko.Heinemann@uk-essen.de (F.M.H.); Peter.Horn@uk-essen.de (P.A.H.); Vera.Rebmann@uk-essen.de (V.R.); 3 Department of Nephrology, University Hospital Essen, University Duisburg-Essen, 45147 Essen, Germany; Anja.Bienholz@uk-essen.de (A.B.); Andreas.Kribben@uk-essen.de (A.K.); 4 Institute for Virology, University Hospital Essen, University Duisburg-Essen, 45147 Essen, Germany; Mirko.Trilling@uk-essen.de

**Keywords:** Human leukocyte antigen-G, HLA-G 3′UTR Polymorphisms, Living Kidney Transplantation, Cytomegalovirus

## Abstract

The expression modulation of the immunosuppressive non-classical Human leukocyte antigen-G (HLA-G) molecule and its soluble isoforms is an immune evasion strategy being deployed by cytomegalovirus (CMV). The +3142 C>G single nucleotide polymorphism (SNP) located within the 3′ untranslated region (3′UTR) is of crucial importance for the regulation of HLA-G expression. Therefore, we analyzed the influence of the +3142 C>G HLA-G SNP on the occurrence of CMV infection in a cohort of 178 living-donor kidney recipients and their 178 corresponding donors. In addition, soluble HLA-G (sHLA-G) levels were quantified before and after transplantation. The presence of the HLA-G +3142 CC genotype in recipients, but not donors of our cohort as along with elevated sHLA-G levels (≥6.1 ng/mL) were associated with higher susceptibility to CMV infection after transplantation. Our results provided evidence that (i) HLA-G is implicated in the establishment of CMV after living-donor kidney transplantation and (ii) recipient HLA-G +3142 CC genotype and sHLA-G concentration levels could represent important predictive risk markers for CMV infection.

## 1. Introduction

Human cytomegalovirus (CMV) is the prototypic member of the β-herpesvirus subfamily that causes widespread, life-long human infections, which are particularly life threatening for immunosuppressed patients after solid organ transplantation [1,2]. In order to escape immune responses, CMV has developed multiple strategies to counteract numerous aspects of the host immune system [3]. In this process, the immunological antagonism of the human leukocyte antigen (HLA) presentation represents an essential mechanism of immune evasion. CMV encodes several gene products (e.g., US2, US3, US6, US10, US11, pp71-UL82, miR-US4-1 etc.) which target classical HLA class I molecules and/or prevent antigen presentation on the surface of infected cells [4,5]. Some of these inhibitory proteins discriminate between classical and non-classical HLA molecules [6], while others affect both types of HLA molecules [7,8]. In this context, the accumulating line of evidences demonstrates that an up-regulated expression of the immune checkpoint molecule HLA-G facilitates viral immune escape [9,10]. HLA-G has the capacity to inhibit immune competent cells thereby modulating the innate and adaptive immune system [11]. It alters natural killer (NK) cell- and T lymphocyte-mediated cytotoxicity and B cell activation by interacting with the corresponding inhibitory receptors immunoglobulin-like transcript 2 (ILT-2) and ILT-4 as well as killer cell immunoglobulin-like receptor 2DL4 (KIR-2DL4) [12,13,14]. Remarkably, similar to the membrane bound forms, the soluble HLA-G facilitates these inhibitory effects [11,15,16,17]. HLA-G is characterized by a low number of allelic variations resulting in limited variability in the protein-encoding region. However, polymorphic sites within the non-coding region such as the 5′ upstream regulatory region (5′URR) and the 3′ untranslated region (3′UTR) are of crucial importance for the regulation of HLA-G expression levels [18,19]. The HLA-G +3142 C>G (rs1063320) single-nucleotide polymorphism (SNP) located within the 3′UTR, affecting the binding site for microRNAs and thereby influencing the expression levels is of particular relevance [20].

In kidney transplantation, CMV reactivation remains one of the most common infectious complications despite the availability of generally effective antiviral therapies [2]. Enhanced HLA-G expression has been associated with allograft tolerance after kidney transplantation [11]. Studies aiming to demonstrate the relevance of HLA-G on the occurrence of CMV infection are limited in transplantation setting and mostly exclusively focused on the recipient HLA-G genotype [21,22]. Considering the immune inhibitory features of HLA-G, differences in recipient and donor HLA-G +3142 C>G polymorphism induce altered protein expression levels, and therefore may possibly have implications on the occurrence of CMV replication during the first year of living-donor kidney transplantation. To address this question, the recipient and the donor HLA-G +3142 C>G SNP was genotyped, in addition to the quantification of pre- as well as post-transplant sHLA-G concentrations. The results were correlated with the clinical outcome in terms of allograft loss and the occurrence of CMV infection.

## 2. Results

We found that the incidence of CMV infection was significantly higher among living-donor kidney transplant recipients carrying at least one +3142 C allele and the homozygous +3142 CC genotype, compared to non-carriers (*p* < 0.0462 and 0.0394, respectively, Table 1A).

**Figure 1:**
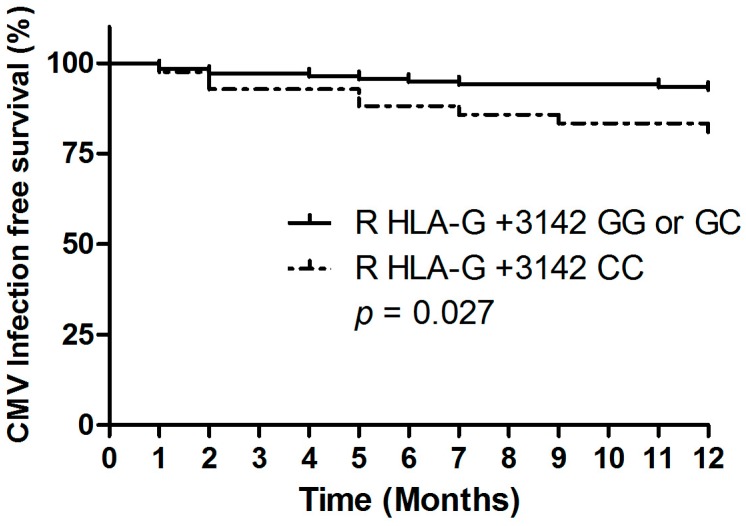
Association between the transplant recipient HLA-G +3142 C>G polymorphism and CMV infection during first year of living-donor kidney transplantation. Recipients with a HLA-G +3142 CC genotype had a significantly increased likelihood of CMV infection.

**Figure 2:**
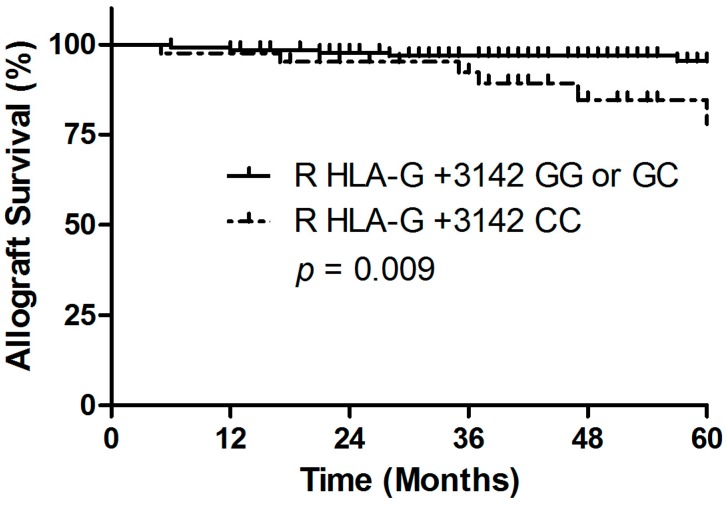
Association between the transplant recipient HLA-G +3142 C>G polymorphism and 5 year allograft survival. Recipients with a HLA-G +3142 CC genotype had a significantly reduced allograft survival.

Taking into account the time course, the results of the Kaplan-Meier plot analysis combined with the log-rank test indicate that the development of CMV during the first year after living-donor kidney transplantation was significantly higher among recipients with a homozygous +3142 CC genotype (*p* = 0.027; Hazard Ratio (HR) 2.7; 95% confidence interval (95% CI): 1.0–6.9, Figure 1), compared to +3142 G allele carriers (i.e., homozygous +3142 GG and heterozygous +3142 CG). Interestingly, we found that the recipient homozygous +3142 CC genotype was associated with an increased risk of 5-year allograft loss (*p* = 0.009, HR 4.3; 95% CI: 1.3–14.2, Figure 2 and Table 2A). The recipient HLA-G +3142 C>G genotype was not associated with acute cellular rejection within the first year after living-donor kidney transplant (data not shown).

With respect to the HLA-G +3142 C>G donor genotype, neither it was associated with occurrence of CMV infection (*p* = 0.77, HR 0.85; 95% CI: 0.28–2.59; Table 1B) or with acute cellular rejection (*p* = 0.39; HR 0.7; 95% CI: 0.3–1.6), while there was a borderline significance with allograft loss (*p* = 0.068; HR 2.9; 95% CI: 0.8–9.5; Table 2B). There was no association with outcome parameters and presence of the HLA-G +3142 C>G mismatch.

Soluble HLA-G molecules are of clinical relevance and exhibit equal immunosuppressive properties as their membrane bund counterparts [17]. In an attempt to identify a predictive marker for CMV infection based on sHLA-G levels, we quantified soluble molecule concentrations in patient sera. In contrast to previous studies that linked levels of sHLA-G to the HLA-G +3142 C>G SNP [19] there was no significant association in our cohort (data not shown).

**Figure 3:**
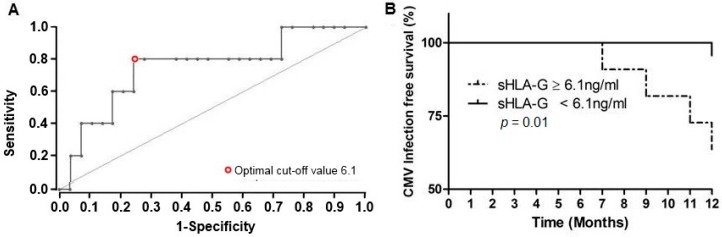
The correlation between plasma levels of soluble human leukocyte antigen-G (sHLA-G) and the occurrence of CMV viremia during the first year of living-donor kidney transplantation. (**A**) Receiver operating characteristic curve-based stratification was performed to predict CMV-infection free survival based on sHLA-G levels. The red open circle indicates the optimal cut-off value with a sensitivity of 80.0% and a specificity of 75.8%; black dots represent differing cut-off values (**B**) patients with higher sHLA-G levels (≥6.1 ng/mL) had a significantly increased likelihood of CMV infection compared to patients with low sHLA-G levels (<6.1 ng/mL).

The sHLA-G levels prior kidney transplantation were significantly elevated in recipients exhibiting productive CMV replication after transplantation compared to those with no viral replication (38.9 ± 13.0 ng/mL vs. 25.1 ± 1.74 ng/mL; *p* = 0.04). After transplantation, the sHLA-G levels dramatically decreased (sHLA-G before 26.8 ± 16.7 ng/mL vs. after transplantation 6.1 ± 4.8 ng/mL; *p* = 0.0002). Soluble HLA-G levels remained nearly twice as high in recipients with CMV reactivation without reaching the level of significance (10.32 ± 1.73 vs. 5.4 ± 0.9 ng/mL; *p* = 0.082). To evaluate whether post-transplant sHLA-G levels can help to identify patients with increased risk of CMV after transplantation, the ROC analysis was performed (Figure 3A). Using an optimal cut-off value of 6.1 ng/mL (AUC = 0.75, sensitivity: 80.0%, specificity: 75.8%), Kaplan-Meier curve analyses combined with the log-rank test revealed that sHLA-G concentrations were significantly associated with an increased occurrence of CMV within the first year after transplant (*p* = 0.010; HR 10.1, 95% CI: 1.1–90.8; Figure 3B).

## 3. Discussion

HLA-G is a naturally occurring immune suppressive molecule [23]. Its surface expression is physiologically restricted to the maternal-fetal interface and to immune privileged adult tissues. However, secreted soluble forms of HLA-G are detectable in a variety of body fluids [24] The clinical implications of the differential modulation of HLA-G gene expression by the regulatory polymorphism within the 3′UTR has been emphasized by a number of studies in a wide range of pathological conditions [11,13,25]. Many reports highlighted the importance of HLA-G either in viral infections or in solid allograft acceptance after transplantation [26,27,28,29], though only a minority focused on the role of HLA-G for CMV infection in an allogeneic setting after transplantation [21,22,30]. In a cohort of living-donor kidney transplant pairs, we were able to uncover that (i) the recipient HLA-G +3142 CC genotype is associated with CMV infection within the first year after transplantation and to the five year allograft loss; (ii) donor HLA-G +3142 C>G polymorphism had no impact on allograft outcome; and (iii) elevated sHLA-G serum concentrations might be useful to discriminate recipients with an occurrence of CMV, but do not predict acute rejection.

There are strong evidences that CMV infection after transplantation promotes inflammation, vasculopathy and cellular rejection of an allograft which impacts on allograft survival [2,31,32,33,34]. Considering the negative impact of CMV infection on allograft outcome the identification of predictive markers for detection of CMV prone transplant recipients benefiting from a more intense monitoring or alternative therapeutic approach is urgently needed. While in healthy individuals most CMV infections are asymptomatic, life-threatening diseases occur under the influence of immunosuppressive regimens in the context of allograft transplantation. Implementing prophylactic antiviral therapy has reduced the occurrence of early CMV disease, but the development of late-onset disease and drug resistance is increasingly recognized [2]. In this context, HLA-G +3142 CC recipient genotype constitutes a promising genetic candidate marker for the identification of CMV prone potential recipients even before kidney transplantation. The functional polymorphism +3142 C>G is of specific interest as it has been shown that the C allelic variation is associated with enhanced HLA-G protein expression [11]. Our results are strengthened by the few previous reports clearly outlining the influence of the HLA-G genotype to CMV-susceptibility [21,22,30]. Considering the various immune suppressive functions mediated by HLA-G, the HLA-G genotype has an impact on level of HLA-G expression and thus provides the background for facilitating the CMV escape mechanism [11].

In our cohort, the presence of the HLA-G CC recipient genotype was not only associated with occurrence of CMV infection but also with reduced allograft survival. It is noteworthy that chronic kidney allograft loss is attributed to multiple immune as well as non-immune injuries, against a background of various donor- and recipient-derived risk factors. In this setting, immune responses directed against donor antigens leading to allograft rejection play an eminent role. In our cohort the co-incidence of CMV infection occurred in 4 out of 35 acute rejection episodes. However, we did not find any association between recipient HLA-G +3142 CC genotype and occurrence of rejection. Acute rejection represents an immunological inflammation that occurs within the microenvironment of the allograft, while CMV infection may comprise a located as well as systemic disease. It is reasonable that the establishment of systemic CMV viremia is dominantly influenced by the recipient specific genetic characteristics whereas in cellular rejection both, recipient- and donor-derived factors influence alloimmune and inflammatory responses. The impact of the donor HLA-G genotype in solid organ transplantation has only been investigated in one liver transplantation study supporting our findings on the low influence of the donor HLA-G genotype on transplantation-outcome [30].

Of importance, there was a significant relationship between post-transplant elevated plasma sHLA-G levels and CMV occurrence. This result suggests that high levels of HLA-G promote susceptibility to viral infections. Nevertheless, no association between certain HLA-G +3142 C>G genotypes and soluble HLA-G levels was observed, which can be partly attributed to the low number of plasma samples available, but also to a variety of additional mechanistic factors affecting soluble molecule concentrations.

Taken together, in the context of transplantation the immunosuppressive checkpoint molecule HLA-G plays an eminent role and may discrepantly affect transplant outcome: while on the one hand HLA-G is involved in tolerogenic responses and high levels of HLA-G are associated with better allograft acceptance [11], on the other hand HLA-G promotes viral immune evasion and as showed in our study HLA-G expression is significantly increased during CMV infection [9,10]. Thus, the immunosuppressive properties of HLA-G create an anti-inflammatory environment and in doing so, contribute to increased susceptibility to CMV infection. Although the precise mechanisms contributing to the immunological hazards of CMV infection after transplantation have not been fully elucidated, the results of clinical studies clearly identify the presence of CMV infection as a risk factor for allograft rejection and loss [2,31,32,33,34]. Therefore, enhanced HLA-G expression upon CMV infection in the allogeneic setting of transplantation may indirectly contribute and predict a worse transplant outcome.

In summary, the results of our study suggest that the +3142 CC recipient genotype and enhanced soluble HLA-G levels are associated with a greater susceptibility to CMV infection after living-donor kidney transplantation. Thus, the HLA-G +3142 C>G polymorphism seems to represent a promising novel genetic biomarker for CMV infection after transplantation that is worth of being verified in future studies.

## 4. Materials and Methods

### 4.1. Study Population and Outcome Parameters

In total, 178 living-donor kidney transplant recipients and their 178 corresponding donors from the transplant program at the University Hospital Essen, Germany, were enrolled in this study. Occurrence of CMV infection was monitored during the first year after transplantation. The following clinical data were collected from electronic patient records: demographic and transplant-related characteristics of recipient and donor, underlying renal disease, CMV serostatus of recipient and donor, incidence of first episode of CMV viremia or disease within 12 months after transplant, and biopsy-proven acute cellular rejection within 12 months after transplant and 5 year allograft loss. CMV high risk patients (grafts derived from CMV-seropositive donor [D+] transplanted in CMV-seronegative recipients [R-] *n* = 38) and patients with a lymphocyte-depleting induction therapy received CMV prophylaxis for 100 days before the end of 2011. Because of emerging data suggesting that the incidence of CMV infection was lower among patients receiving a prolonged antiviral prophylaxis in 2012 the duration of the prophylactic CMV regimen was prolonged to 200 days for the high-risk population [35]. All other patients were pre-emptively monitored for CMV viremia weekly for the period of 3 months and afterwards monthly. CMV infection or disease was classified according to recent recommendations [36]: CMV infection was defined as CMV viremia (polymerase chain reaction >400 copies/mL or >1/100 pp65/pUL83 antigen positive cells); CMV disease was defined as CMV viremia in combination with attributable symptoms, such as fever, malaise, leukopenia, thrombocytopenia, or elevation of liver enzymes. CMV complications were analyzed within the first 12 months after transplantation. Incidence of first episode of clinically significant CMV viremia/disease within the 12 months follow-up was 10% (*N* = 18).

Additional clinical outcome parameters of this study were biopsy-proven rejection events within the first year and five year allograft loss. Allograft loss was defined as return to dialysis or estimated Glomerular filtration rate <15 mL/min/1.73 m². Detailed cohort characteristics are shown in Table 3.

Signed informed consent was obtained from all patients in accordance with the Declaration of Helsinki, and the ethics committee of the University Hospital Essen approved the study (12-5312-BO; April 2013) .

### 4.2. HLA-G +3142 C>G SNP Typing and Soluble HLA-G Enzyme-Linked Immunosorbent Assay (ELISA)

Genotyping of the HLA-G +3142 C>G SNP was performed as previously described [37]. Briefly, for +3142 C>G polymorphism 50  ng of genomic DNA of genomic DNA was amplified in a reaction tube, with final concentrations as follows: PCR buffer 1.5  mM MgCl2; 1.8  mM of dNTP; Taq DNA polymerase and 100  pmol of each primer (GMIRNAF–5′-CATGCTGAACTGCATTCCTTCC-3′, GMIRNAR–5′-CTGGTGGGACAAGGTTCTACTG-3′). Thermocycling conditions were as follows: 94 °C for 5  min; 32  cycles of 94 °C for 30  s, 65.5 °C for 30  s and 72 °C for 60  s followed by a final extension step at 72 °C for 5  min. The amplified PCR products were cleaved with 3 U of the restriction enzyme BaeGI (New England Biolabs, Inc., Ipswich, MA, USA), according to the manufacturer’s instructions. RFLP products were analyzed by electrophoresis in a 2% (*w*/*v*) agarose gel stained with ethidium bromide, with amplicon sizes of 406  bp for the C allele and 316 and 90 bp for the G allele. The distribution of HLA-G +3142 C>G SNP genotypes and alleles for recipients and donors is summarized in Table 3. The HLA-G +3412 genotype and allele frequencies were similar among recipients and donor. The observed allelic distribution was in accordance with expectations indicated by the Hardy-Weinberg equilibrium (*p* > 0.05).

A sHLA-G-specific enzyme-linked immunosorbent assay (ELISA) was performed as previously described [38]. Briefly, plasma samples were used in a dilution of 1:2 in PBS. Purified HLA-G5 served as standard reagent. The sHLA-G levels were determined by four-parameter curve fitting. The ELISA detection limit of sHLA-G was 0.25 ng/mL. Plasma soluble HLA-G levels were determined in 57 patients before and consecutively in 34 recipients in a longitudinal follow-up after kidney transplantation.

### 4.3. Statistical Analysis

Baseline characteristics of donors and recipients were compared with two-sided Fisher’s exact test or the Wilcoxon rank-sum test, as appropriate. The occurrence of CMV infection was estimated by the method of Kaplan-Meier and survival curves were compared using the log-rank test. We used multivariate Cox proportional hazards modeling to assess the risk of CMV infection after transplantation. A two-sided *p*-value of 0.05 or lower was considered statistically significant.

## Figures and Tables

**Table 1:** The genotype distribution and allele frequencies of the +3142 C>G (rs1063320) gene polymorphism in living-donor kidney transplant recipients; (**A**) and corresponding donors (**B**) with respect to cytomegalovirus (CMV) infection.

**(A) Recipient**	**CMV Infection *N* = 18**	**No CMV Infection *N* = 160**	***p* Value**	**OR**	**CI (95%)**
C/C	8 (44.4%)	34 (21.3%)	0.0394	2.965	1.082–8.092
C/G	5 (27.8%)	80 (50%)	0.0855	0.384	0.131–1.129
G/G	5 (27.8%)	46 (28.7%)	1	1.037	0.35–3.07
Allele					
C	26	148	0.0462	2.01	1.028–3.947
G	15	172			
**(B) Donor**	**CMV Infection *N* = 18**	**No CMV Infection *N* = 160**	***p*** **Value**	**OR**	**CI (95%)**
C/C	4 (22.2%)	41 (25.6%)	1	0.82	0.26–2.66
C/G	8 (44.4%)	85 (53.1%)	0.62	0.7059	0.26–1.882
G/G	6 (33.3%)	34 (21.3%)	0.24	1.85	0.64–5.3
Allele					
C	16	167	0.386	0.732	0.366–1.46
G	20	153			

**Table 2:** The genotype distribution and allele frequencies of the +3142 C>G (rs1063320) gene polymorphism in living-donor kidney transplant recipients (**A**) and the corresponding donors; (**B**) with respect to allograft loss.

**(A) Recipient**	**Graft Loss *N* = 11**	**No Allograft Loss *N* = 167**	***p* Value**	**OR**	**CI (95%)**
C/C	6 (54.5%)	36 (21.6%)	0.022	4.37	1.26–15.14
C/G	4 (36.4%)	81 (48.5%)	0.54	0.61	0.17–2.15
G/G	1 (9.1%)	50 (29.9%)	0.29	0.25	0.03–2.03
Allele					
C	16	153	0.0158	3.155	1.204–8.263
G	6	181			
**(B) Donor**	**Graft Loss *N* = 11**	**No Allograft Loss *N* = 167**	***p* Value**	**OR**	**CI (95%)**
C/C	5	40	0.125	3.175	0.87–11.5
C/G	5	88	0.76	0.75	0.22–2.54
G/G	1	39	0.46	0.33	0.04–2.65
Allele					
C	15	168	0.125	2.11	0.84–5.33
G	7	166			

**Table 3:** Demographic and clinical characteristics of living-donor transplant recipients and corresponding donors at baseline. Abbreviations are as follows: y: years; SD: standard deviation, HLA: human leukocyte antigen, CMV: cytomegalovirus, R: recipient, D: donor, KTx: kidney transplant.

	Total	HLA-G +3142 GG or GC carrier	HLA-G +3142 CC carrier	*p* Value HLA-G +3142 GG/GC vs. CC
Recipient	*N* = 178	*N* = 136	*N* = 42	
Gender (men/women)	106/72	85/51	21/21	0.15
Age (y ± SD)	41.9 ± 15.9	41.1 ± 15.6	44.6 ± 9.5	0.96
CMV positive recipient (R+)	91	73	18	0.22
Donor	*N* = 178	*N* = 133	*N* = 45	
Gender (men/women)	71/107	56/77	15/30	0.29
Age (y ± SD)	51.4 ± 9.7	50.8 ± 9.6	53.0 ± 9.9	0.19
CMV positive donor (D+)	97	70	27	0.39
Cause of end-stage renal disease				
Diabetes mellitus	9	6	3	0.44
Chronic glomerulonephritis	56	42	14	0.85
Polycystic kidney disease	24	18	6	0.80
Other or unknown	89	70	19	0.60
Transplant related characteristics				
Mean cold ischemia time (minutes ± SD)	133.5 ± 49.4	131 ± 51.2	140.6 ± 42.9	0.67
Lymphocyte-depleting induction therapy (yes/no)	12/166	8/128	4/38	0.41
AB0 incompatible transplant (yes/no)	20/158	14/122	6/36	0.47
HLA A, B mismatches (mean ± SD)	2.0 ± 1.2	2.01 ± 1.15	2.0 ± 1.1	0.78
HLA-DR mismatch (mean ± SD)	1.1 ± 0.7	1.1 ± 0.7	1.2 ± 0.6	0.85
Transplantation outcome parameters				
CMV infection first year after KTx total group (yes/no)	21/157	12/124	9/33	0.027
5 year Allograft loss (yes/no)	11/167	5/131	6/36	0.013
Acute cellular rejection (yes/no)	36/142	28/108	8/34	0.82

## Abbreviations

HLA	Human leukocyte antigen
sHLA-G	(Soluble) Human leukocyte antigen-G
CMV	Cytomegalovirus
SNP	Single Nucleotid Polymorphism
3′UTR	3′ untranslated region
NK cell	Natural killer cell
ILT	Immunoglobulin-like transcript
KIR	Killer cell immunoglobulin-like receptor
5′URR	5′ upstream regulatory region
HR	Hazard Ratio
95% CI	95% Confidence interval
R	Recipient
D	Donor
US	Ultra short
eGFR	Estimated Glomerular Filtration Rate
ELISA	Enzyme-linked immunosorbent assay
